# Curcumin ameliorated low dose-Bisphenol A induced gastric toxicity in adult albino rats

**DOI:** 10.1038/s41598-022-14158-1

**Published:** 2022-06-17

**Authors:** Omnia Ibrahim Ismail, Manal Mahmoud Samy El-Meligy

**Affiliations:** 1 Human Anatomy and Embryology Department, Faculty of Medicine, Assiut University, Assiut, Egypt; 2 Human Anatomy and Embryology Department, Faculty of Medicine, Suez University, Suez, Egypt

**Keywords:** Cell biology, Structural biology, Anatomy

## Abstract

Bisphenol A (BPA) is one of the most common worldwide chemicals involved in the industry of polycarbonate plastics, medical devices, and pharmaceuticals. Forty three-month-old albino rats were randomly classified into four groups. Group Ӏ received a daily corn oil dose (5 mL/kg/ body weight, BW) through a gastric tube for one month, Group ӀӀ received a daily dose of Curcumin (200 mg/kg body weight (B.W.) through a gastric tube for one month, Group ӀӀӀ received a daily dose of BPA (0.5 μg/kg B.W.) through a gastric tube for one month and Group ӀV received concomitant daily doses of Bisphenol A and Curcumin as the regimen described in groups ӀӀ and ӀӀӀ. The rats were sacrificed, and glandular portion of stomach was dissected and processed for light, immunohistochemical and ultrastructural study. BPA induced destructed gastric glands, dilated congested blood vessels, submucosal oedema, decreased PAS-positive reactivity, increased collagen fibres deposition, decrease in the positive BCL2 immunoexpression, increased positive PCNA immunoexpression, reduction in the gastric mucosal height and destructive changes in the enteroendocrine, chief and parietal cells. Curcumin coadministration provoked an obvious improvement in the gastric structure. BPA exposure has toxic effects on the glandular portion of the stomach in rats. Otherwise, Curcumin coadministration has exhibited protective impact on the architecture of the stomach.

## Introduction

Bisphenol A (BPA) [2,2-bis(4-hydroxyphenyl) propane] is a common chemical used globally. BPA is intensely used in the polycarbonate plastic industries and daily use products, such as food packaging, toys, infant feeding bottles, medical devices, and pharmaceuticals. BPA has been getting more attention due to its link to various health problems that develop after direct or indirect human exposure^1^.

BPA molecules leak from food and beverage containers to food and drinks due to the exposure of polycarbonate products to high temperatures or reaction with acidic or basic components^2^. Therefore, BPA and its metabolites have been found in about 92.6% of human bodies^3^.

BPA exposure in humans is primarily through the intake of food and drinking water stored in containers made of BPA and inhalation of BPA-contaminated dust during synthesis^4^. Moreover**,** the main exposure route for BPA is the digestive system in animals and humans^5^.

BPA is absorbed by the gastrointestinal tract, metabolized in the liver into BPA glucuronide, distributed to the blood and tissues through the endocrine system, and excreted via the kidneys in urine^6,7^**.** BPA acts as an agonist, antagonist, or modulator of hormone receptors and has potent estrogenic action^8^.

Furthermore, BPA induces neurological toxicity, degenerative changes in the structure of cardiac and renal tissues, various diseases, such as diabetes, coronary heart disease, endometrial hyperplasia, prostate and breast cancer, reproductive disorders, and liver dysfunction, and suppresses the immune system^9–12^.

Curcumin (*Curcuma longa*) is a natural antioxidant polyphenol used for coloring and flavoring in foods and in ancient Asian medicine. Its ingestion might improve several chronic disorders, such as obesity, diabetes, and hypertension^13^. Moreover, curcumin protects tissues against environmental toxins^14^, and inhibits cell proliferation and tumor growth. Several studies have demonstrated that curcumin possesses antioxidant, immunomodulatory, antiinflammatory, and antiangiogenic properties via its impacts on multiple cell-signaling pathways^15,16^.

Furthermore, as evidence for the dangers of BPA exposure grows, more studies have been conducted to evaluate these dangers on the male and female reproductive systems, with little information available concerning their effect on the stomach. Moreover, several studies have demonstrated the destructive effect of high BPA doses, but the effect of small doses on the histological structure of different organs is controversial. Therefore, this study was designed to analyze the histological and immunohistochemical effects of low-dose BPA on the glandular portion of the stomach in adult albino rats. Furthermore, we determined whether the co-administration of curcumin could treat infections.

## Materials and methods

### Chemicals

Bisphenol A (catalog number: 50-175-0991) and curcumin (catalog number: 501837713) were purchased from Sigma Company, Egypt.

### Animals

Forty female albino rats aged 3 months and weighing 200 ± 30 g were used in this study. The rats were obtained from the Animal House of the Faculty of Medicine, Assiut University. Also, the experiment was approved by the Institutional Ethics Committee of Assiut University (IRB local approval number: 17300686). The rats were housed in separate cages under controlled laboratory conditions in a 12-h light/dark cycle and received a standard rodent diet and water ad libitum.

### Experimental design

The 40 rats were classified randomly into four groups, and each group consisted of ten rats:

- *Group Ӏ (negative control group)* received a daily corn oil dose (5 mL/kg/ body weight, BW) through a gastric tube for one month.
- *Group ӀӀ (positive control group)* received a daily curcumin dose (200 mg/kg BW) through a gastric tube for one month. Curcumin is a bright orange powder dissolved in corn oil^17^**.**
- *Group ӀӀӀ (BPA group)* received a daily dose of BPA (0.5 μg/kg BW) through a gastric tube for one month. BPA is an odorless white crystalline powder that was dissolved in corn oil^18^.
- *Group ӀV (BPA + curcumin group)* received concomitant daily doses of BPA and curcumin as the regimen described in groups ӀӀ and ӀӀӀ.

At the end of the experiment, rats in all groups were weighed and then anesthetized by ether inhalation. Also, an automated balance S/SI-2002 (Fisher Scientific, New York, USA) was used. They were subjected to intracardiac perfusion by normal saline and 0.9% NaCl, then sacrificed. Their stomachs were dissected, rinsed, weighed, and incised along the greater curvature. Finally, specimens from the glandular portion were taken and prepared for light and electron microscopy.

#### Light microscopy

The specimens were fixed in 10% neutral formalin then prepared for paraffin blocks. The blocks were cut into 5-mm sections and stained with hematoxylin and eosin for examining the histological structure^19^**,** periodic acid-Schiff (PAS) for evaluating mucosal glycoprotein production variations in the mucosa of the stomach^20^, and Masson’s Trichrome for detecting collagen fiber deposition^21^**.**

#### Electron microscopy

The specimens were trimmed about 3 mm^**3**^ to allow electrons to cross through the sample. Primary fixation with 2.5% glutaraldehyde + 4% formaldehyde for 2 h at room temperature was conducted; then, distilled water (DH_2_O) was added three times in 10 min for washing. Next, 1% osmium tetroxide was added for 1 h and washed out in DH_2_O three times in 10 min. Finally, a dehydration series by ascending grades of ethanol was applied as follows: 30%, 50%, 70%, 90%, and 100% (each for 10 min).

The tissues were embedded in a pure Epon-Araldite mixture**.** Ultra-microtome, semithin sections (1 µ) were obtained and stained with 2% aqueous toluidine blue and dried on a hot plate at 40 °C. Ultra-thin sections (0.1 µ) were obtained using Leica Ultra-Cut UCT (Deerfield, IL, USA), then mounted on copper grids and stained with uranyl-acetate and lead citrate using a double staining technique^22^. The stained ultra-thin sections were examined using a transmission electron microscope (TEM) (“Jeol” E.M.-100 CX11; Japan) at the Electron Microscopic Unit of Assiut University, Assiut, Egypt.

#### Immunohistochemical study

Gastric paraffin sections were deparaffinized in xylene for 25 min at 60 °C in an oven, then rehydrated with graded alcohol and washed with phosphate-buffered saline (PBS). Then, 0.1% hydrogen peroxide was incubated on the sections for 30 min to block endogenous peroxidase. Also, antigen retrieval was conducted using 10-mM sodium citrate buffer with pH 6.0 (1 :10 dilution) and boiled in a microwave for 25 min. Primary antibodies: anti-rat BCL2 (1:50 dilution; catalog number MA5-11,757; Thermo Fisher Scientific, Lab Vision, USA), PCNA (proliferating cell nuclear antigen; 1:100 dilution; catalog number MA5-11,358; Thermo Fisher Scientific, Lab Vision, USA), and EGFR (epidermal growth factor receptor; 1:100 dilution; catalog number 44-790G; Thermo Fisher Scientific, Lab Vision, USA) were applied on the tissue sections in a wet chamber at 4 °C overnight. The slides were rinsed with PBS three times then incubated with diluted 1:200 secondary goat anti-mouse IgG peroxidase-conjugated antibody in a wet chamber for 30 min at room temperature. Staining was completed by incubation with diaminobenzidine (DAB), resulting in a brown-colored precipitate at the antigen sites. Mayer’s hematoxylin was used as a counterstain followed by dehydration in graded ethanol, clearance with xylene, and mounting with DPX. A positive immunohistochemical reaction appeared as brown stains under a light microscope. Finally, a negative control was generated using normal tissues and omitting the primary antibody, according to^23^. The positive controls for each immunostaining were taken as following: spleen sections for bcl-2, tonsil sections for PCNA and placental sections for EGFR.

#### Morphometric analysis

The gastric mucosal height was measured as a perpendicular distance between the gastric mucosal surface and the muscularis mucosa using 5-mm sections of the stomach. The mucosal height was measured at five approximately equidistant points for each animal in each group using a magnification of × 100 as described by Bauer et al.^24^.

The number of positive immune-expressing PCNA cells at × 1000 magnification was counted using a computerized image analyzer system software (Leica Q 500 MCO; Leica, Wetzlar, Germany) connected to a camera and attached to Leica universal microscope at Human Anatomy and Embryology Department, Faculty of Medicine, Assiut University, Assiut, Egypt and expressed as cell number per field. Five fields within the sections of each animal in each group were evaluated according to Bayomy et al.^25^. The area percent of BCL2 and PCNA immunoreaction as well as BCL2 and PCNA optical density were measured via computerized image analysis software (ImageJ v1.51a) in five fields at × 200 magnification from each slide in different studied groups.

#### Statistical analysis

The data were expressed as mean ± standard deviation. Statistical analysis by one-way analysis of variance followed by Turkey’s post hoc test was performed using SPSS v.13.00 (Chicago, Illinois, USA). Differences between the groups for variance comparison were statistically significant at *P* ≤ 0.05, as reported by^26^.

### Ethics approval and consent to participate

Ethical approval obtained from the Ethical Committee of Faculty of Medicine, Assiut University, Egypt. All methods were performed in accordance with the relevant guidelines and regulations and in compliance with ARRIVE guidelines for the care and use of experimental animals by the committee for the purpose of supervision of Experiment on animals (CPCSEA) and the National institute of Health NIH Protocol. (IRB local approval number: 17300686).

## Results

### General condition and mortality rate

All animals were observed daily for mortality, morbidity and general condition throughout the study and no abnormalities were detected.

### Morphometric and statistical analysis

#### Final body and stomach weights

BPA group showed a significant reduction in the final body weight when compared to the negative and positive control rats. Moreover, BPA + Curcumin group exhibited a higher final body weight relative to BPA group. ANOVA test evaluation revealed that a statistically significant difference was present on comparing all studied groups with *P*-value < 0.0001 (Table 1).

Regarding the stomach weight, BPA group demonstrated a lower value as compared to the negative and positive control groups. Interestingly, with Curcumin administration in group IV an increase in the stomach weight was noticed in comparison to BPA group. The stomach weight showed a statistically significant difference on comparing all studied groups with P-value < 0.0001 (Table 1).

#### Gastric mucosal height

BPA group exhibited a reduction in the gastric mucosal height compared to both negative and positive control groups. Meanwhile, BPA + Curcumin group showed a higher value in comparison to BPA group. Moreover, a statistically significant difference appeared on comparing the gastric mucosal heights of all studied groups with *P*-value < 0.0001 (Table 1).

#### The number of positive immunoexpression PCNA cells

BPA group showed a statistically significant increase in the number of positive immunoexpression PCNA cells per 18,338.4 µm^2^ compared to both negative and positive control groups. Meanwhile, BPA + Curcumin group showed a lower significant value in comparison to BPA group. There was a statistically significant difference on comparing the numbers of positive immunoexpression PCNA cells in all studied groups with *P*-value < 0.0001 (Table 1).

**Table 1:** Final body and stomach weights, gastric mucosal heights, and numbers of positive immunoexpression PCNA cells of the studied groups.

Parameters	Body weight(g)	Stomach weight(g)	Gastric mucosal height(µm)	Number of positive immunoexpression PCNA cells
**Groups**				
Group I	242.9 ± 2.41	1.607 ± 0.11	924.8 ± 58.90	4.30 ± 1.2
Group II	243.6 ± 2.28	1.495 ± 0.12^a^	1001 ± 95.95	4.40 ± 1.2
Group III	232.3 ± 1.70^a,^^b^	1.176 ± 0.09^a,^^b^	600.3 ± 66.86^a,^^b^	13.95 ± 1.4^a,^^b^
Group VI	237.6 ± 2.23^a,^^b,c^	1.291 ± 0.13^a,^^b,c^	695.7 ± 77.45^a,^^b,c^	8.85 ± 1.9^a,^^b,c^
*P*-value	< 0.0001*	< 0.0001*	< 0.0001*	< 0.0001*

Data are represented as Mean ± SD (n = 20)

*means statistically significant difference.

^a^Statistically significant as compared with group I, *P* < 0.05.

^b^Statistically significant as compared with group II, *P* < 0.05.

^c^Statistically significant as compared with group III, *P* < 0.05.

### Light microscopic study

#### H&E stain

**Figure 1:**
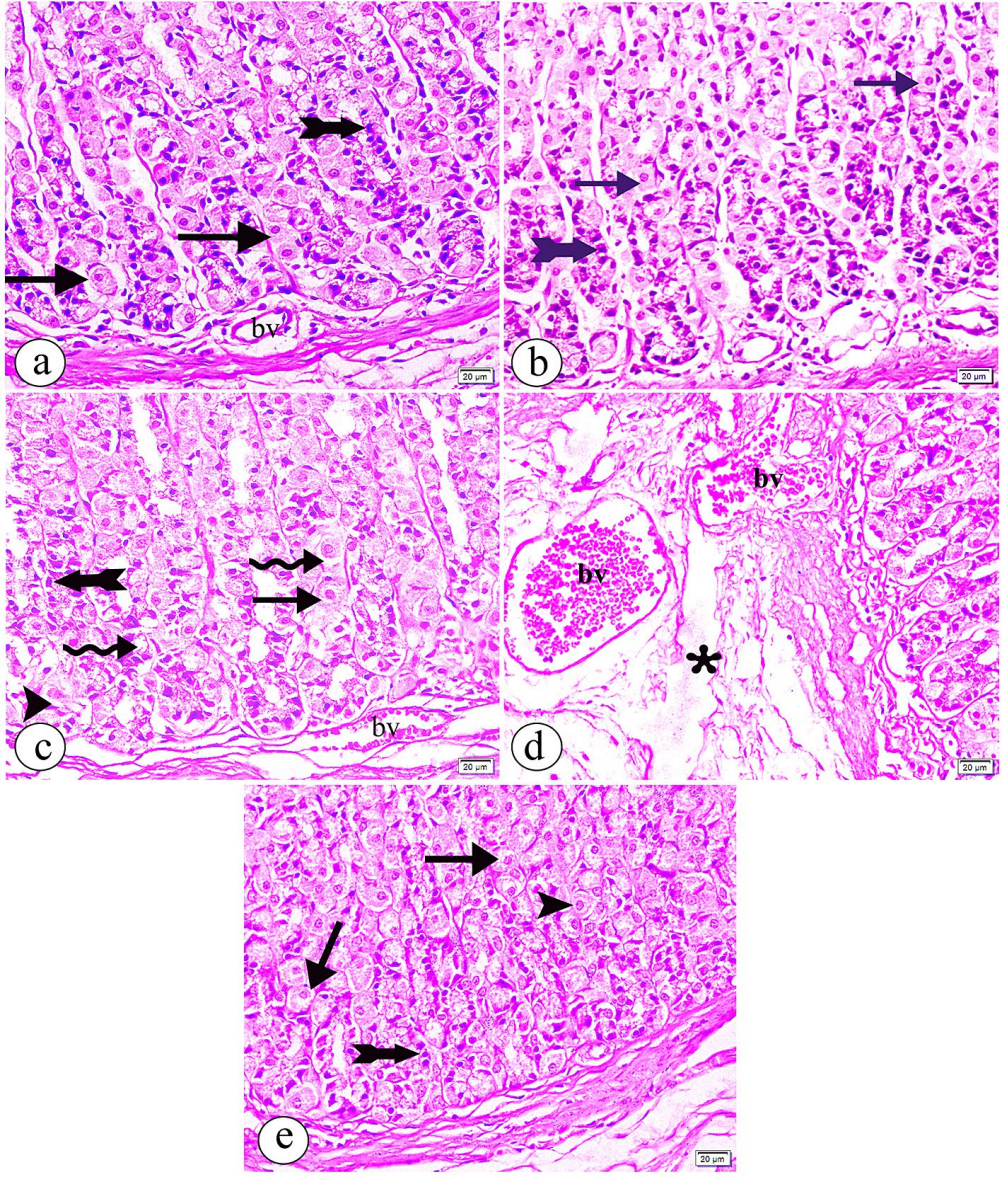
The photomicrographs of the sections in the glandular portion of the stomach in (**a**) the group Ӏ showing the gastric mucosa composed of the parietal cells (arrow) having a central spherical nucleus and an acidophilic cytoplasm. Small Chief cells (tailed arrow) have basal nuclei and an apical blue-purple stained cytoplasm. (**b**) the group ӀӀ showing the gastric mucosa composed of the parietal cells (arrow) and Chief cells (tailed arrow) that appear more or less than in the group Ӏ. (**c**) the group ӀӀӀ showing destructed gastric glands (arrow head) with vacuolations (curved arrow) and dilated congested blood vessels (bv). The parietal cells have dense nuclei and cytoplasm(arrow). Chief cells have a vacuolated cytoplasm (tailed arrow). (**d**) the group ӀӀӀ showing dilated congested blood vessels (bv). Submucosal oedema is observed (asterisk). (**e**) the group ӀV showing some parietal cells have a vacuolated cytoplasm (arrow). Other parietal cells (arrowhead) and Chief cells (tailed arrow) appear more or less than normal. Hx & E, × 400, scale bar = 20 µm).

Light microscopic exanimation of H&E-stained sections demonstrated that the glandular portion of the stomach in both groups Ӏ and ӀӀ was composed of tubular multiple branched glands. The parietal cells appeared with central nuclei and a pale acidophilic cytoplasm. Chief cells were small, located in the basal parts of the glands and had basal nuclei and an apical blue-purple stained cytoplasm (Fig. 1a, b, respectively). In contrast, when Bisphenol A was administered, destroyed gastric glands were found in the group. The parietal cells had dense nuclei and cytoplasm. Chief cells revealed vacuolations in their cytoplasm (Fig. 1c). Also, dilated, congested blood vessels and submucosal oedema were observed (Fig. 1d). Evaluation of the sections in group ӀV revealed that some parietal cells had vacuolated cytoplasm. On the contrary, other parietal cells and Chief cells appeared more or less than normal (Fig. 1e).

#### Periodic acid schiff stain (PAS)

**Figure 2:**
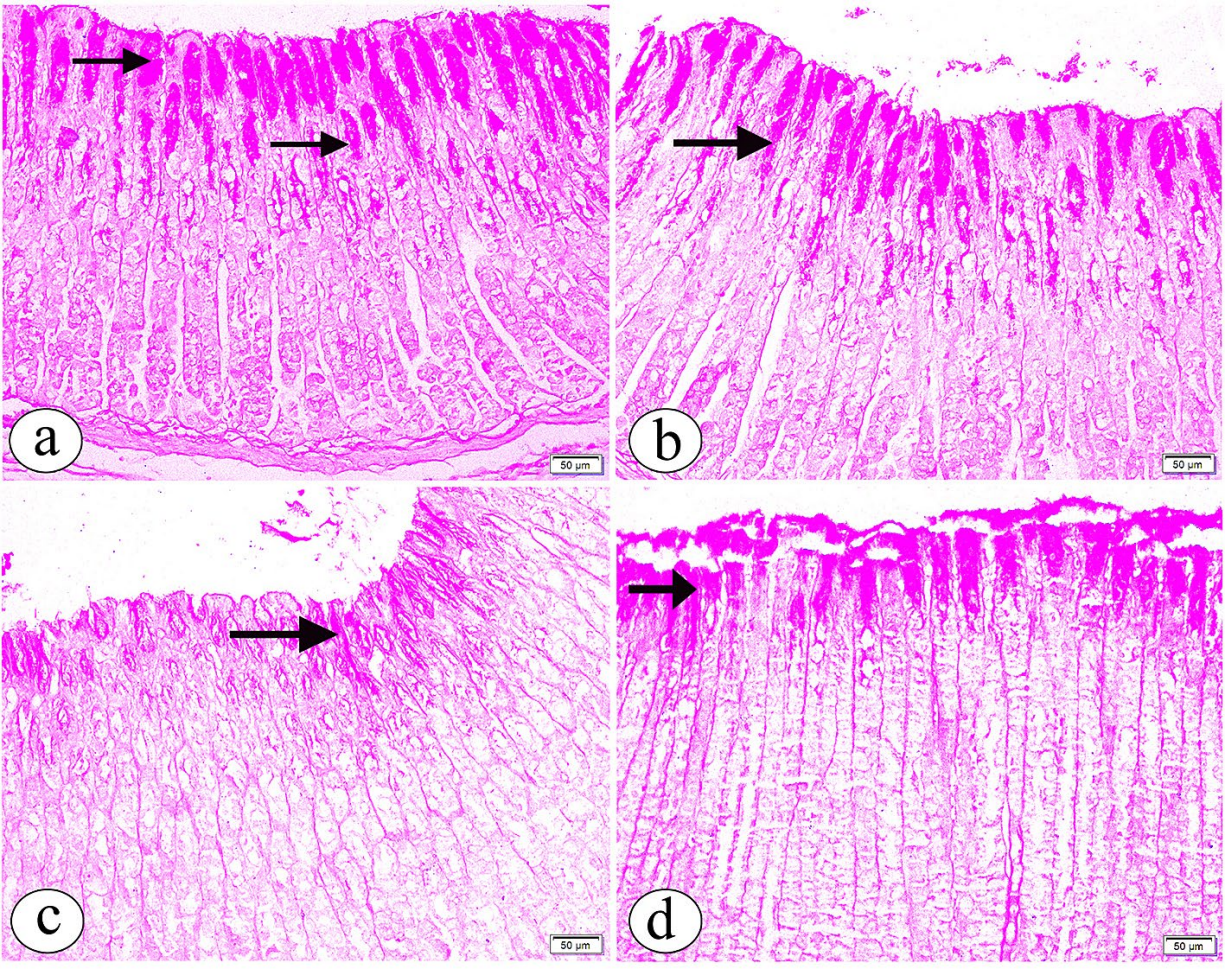
The photomicrographs of the sections in the glandular portion of the stomach in (**a**) the group Ӏ showing a strong positive Periodic Acid Schiff (PAS) reaction in the lining epithelium and neck cells (arrow) is noticed. (**b**) the group ӀӀ showing a strong positive PAS reaction (arrow) more or less than in group Ӏ. (**c**) the group ӀӀӀ showing a decrease in PAS reaction in the lining epithelium and neck cells (arrow). (**d**) the group ӀV showing a restoration of the positive PAS reaction in the lining epithelium and neck cells (arrow). (PAS, × 200, scale bar = 50 µm).

In both groups Ӏ and ӀӀ, PAS-stained sections demonstrated a strong positive reaction in the lining epithelium and neck cells of the glandular portion of the stomach (Fig. 2a, b, respectively). Interestingly, with Bisphenol A administration, a decreased PAS-positive reactivity was observed (Fig. 2c). With coadministration of Curcumin, PAS-stained sections showed a restoration of the positive PAS reaction (Fig. 2d).

#### Masson’s trichrome stain

**Figure 3:**
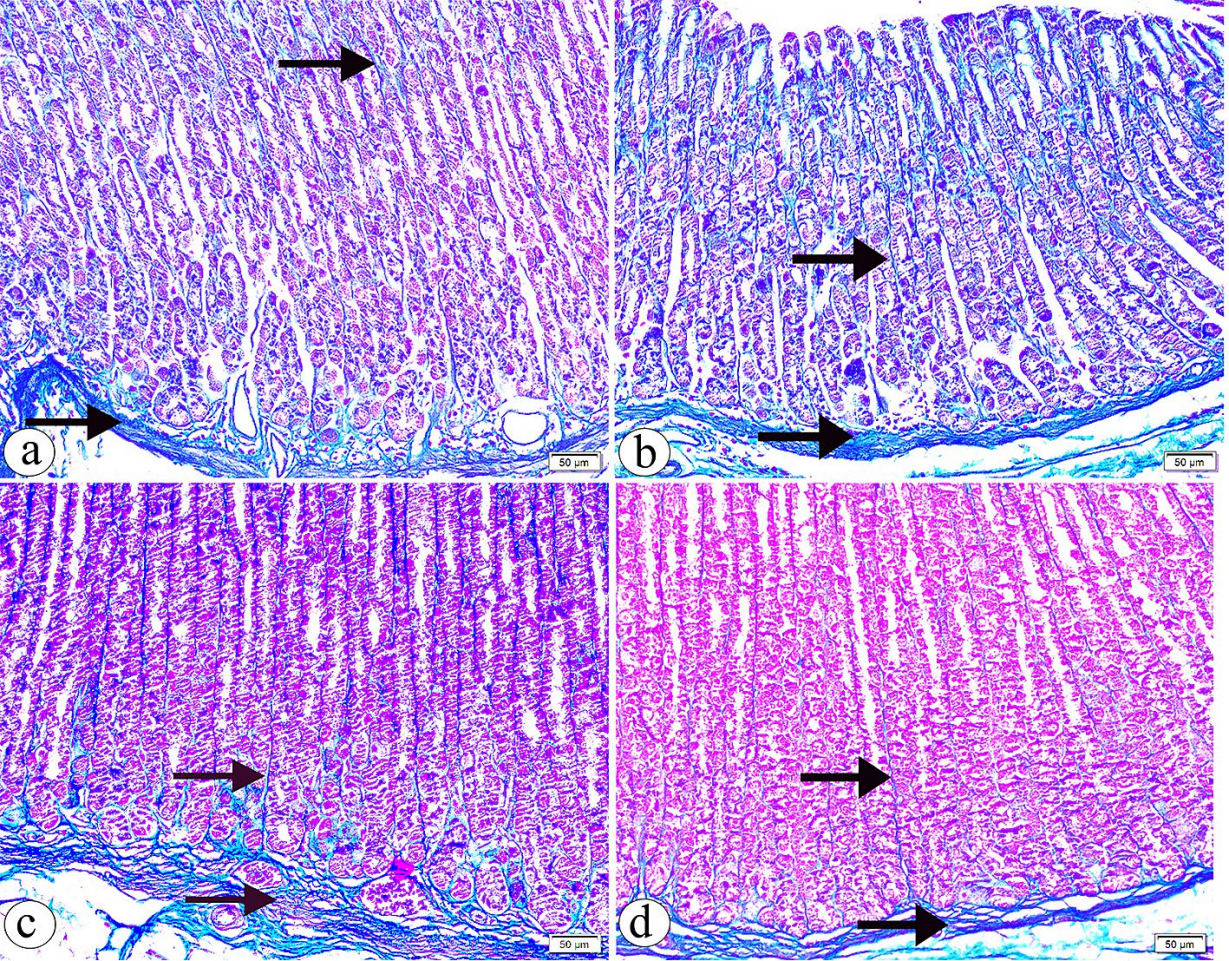
The photomicrographs of the sections in the glandular portion of the stomach in (**a**) the group Ӏ showing a normal distribution of the collagen fibres among the bases of the gastric glands, in the lamina propria and interstitial between the glands (arrow). (**b**) the group ӀӀ showing the collagen fibres distribution (arrow) more or less than the group Ӏ. (**c**) the group ӀӀӀ showing an increased collagen fibres deposition (arrow). (**d**) the group ӀV showing a minimal interstitial collagen fibres deposition (arrow). (Masson’s Trichrome, × 200, scale bar = 50 µm).

Gastric sections showed the normal distribution of the collagen fibres among the bases of the gastric glands, in the lamina propria and interstitial between the glands in both groups Ӏ and ӀӀ (Fig. 3a, b, respectively). However, an increased collagen fibres deposition was detected in group ӀӀӀ (Fig. 3c). Surprisingly, the minimal interstitial collagen fibres deposition was noticed in group ӀV (Fig. 3d).

### Immunohistochemical study

**Figure 4:**
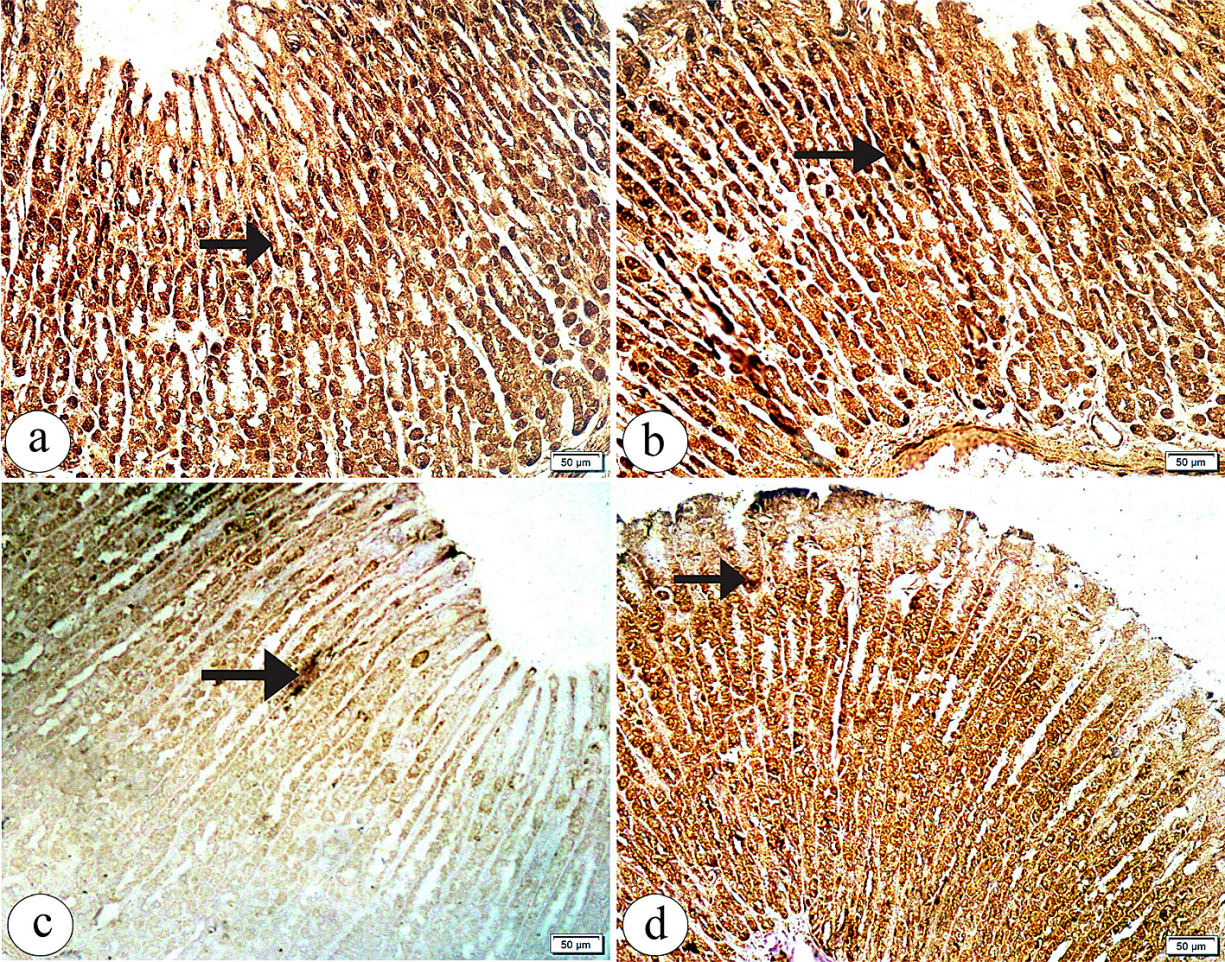
The photomicrographs of the sections in the glandular portion of the stomach in (**a**) the group Ӏ showing a positive BCL2 immunoexpression (arrow) in the gastric glands. (**b**) the group ӀӀ showing a positive BCL2 immunoexpression (arrow) more or less than the group Ӏ. (**c**) the group ӀӀӀ showing an apparent decrease in the positive BCL2 immunoexpression (arrow) as compared to the group Ӏ (**d**) the group ӀV showing an apparent increase in the positive BCL2 immunoexpression (arrow) as compared to the group ӀӀӀ. (BCL2 immunohistochemical staining × 200, Scale bar = 50 µm).

Group Ӏ showed a positive BCL2 immunoexpression in the gastric glands (Fig. 4a). Also, group ӀӀ showed a positive BCL2 immunoexpression more or less than group Ӏ (Fig. 4b). In contrast, BPA treated group showed an apparent decrease in the positive BCL2 immunoexpression as compared to group Ӏ (Fig. 4c). Conversely, group ӀV demonstrated an obvious increase in the positive BCL2 immunoexpression in comparison to group ӀӀӀ (Fig. 4d). The morphometric analysis confirmed that there was a significant decrease in the area percent of BCL2 immunoreaction and BCL2 optical density with BPA treatment as compared to control group. On the other hand, with coadministration of Curcumin a significant increase in the area percent of BCL2 immunoreaction and BCL2 optical density as compared to group ӀӀӀ (as showed in Table 2).

**Table 2:** The area percent of BCL2 immunoreaction (BCL2 area %), BCL2 optical density (BCL2 OD), area percent of PCNA immunoreaction (PCNA area %) and PCNA optical density (PCNA OD) of the studied groups.

Parameters	BCL2 area %	BCL2 OD	PCNA area %	PCNA OD
**Groups**				
Group I	29.47 ± 1.71	0.23 ± 0.020	6.36 ± 0.86	0.079 ± 0.010
Group II	30.71 ± 2.02	0.22 ± 0.01	6.26 ± 0.82	0.080 ± 0.011
Group III	7.86 ± 1.22^a,b^	0.12 ± 0.006^a,b^	10.69 ± 1.14^a,b^	0.114 ± 0.090
Group VI	22.07 ± 2.04^a,b,c^	0.24 ± 0.010^b,c^	8.29 ± 0.75^a,b,c^	0.078 ± 0.012
*P*-value	< 0.0001*	< 0.0001*	< 0.0001*	= 0.55^ ns^

Data are represented as Mean ± SD (n = 20).

*Means statistically significant difference.

^ns^ means non-significant difference.

^a^Statistically significant as compared with group I, *P* < 0.05.

^b^Statistically significant as compared with group II, *P* < 0.05.

^c^Statistically significant as compared with group III, *P* < 0.05.

**Figure 5:**
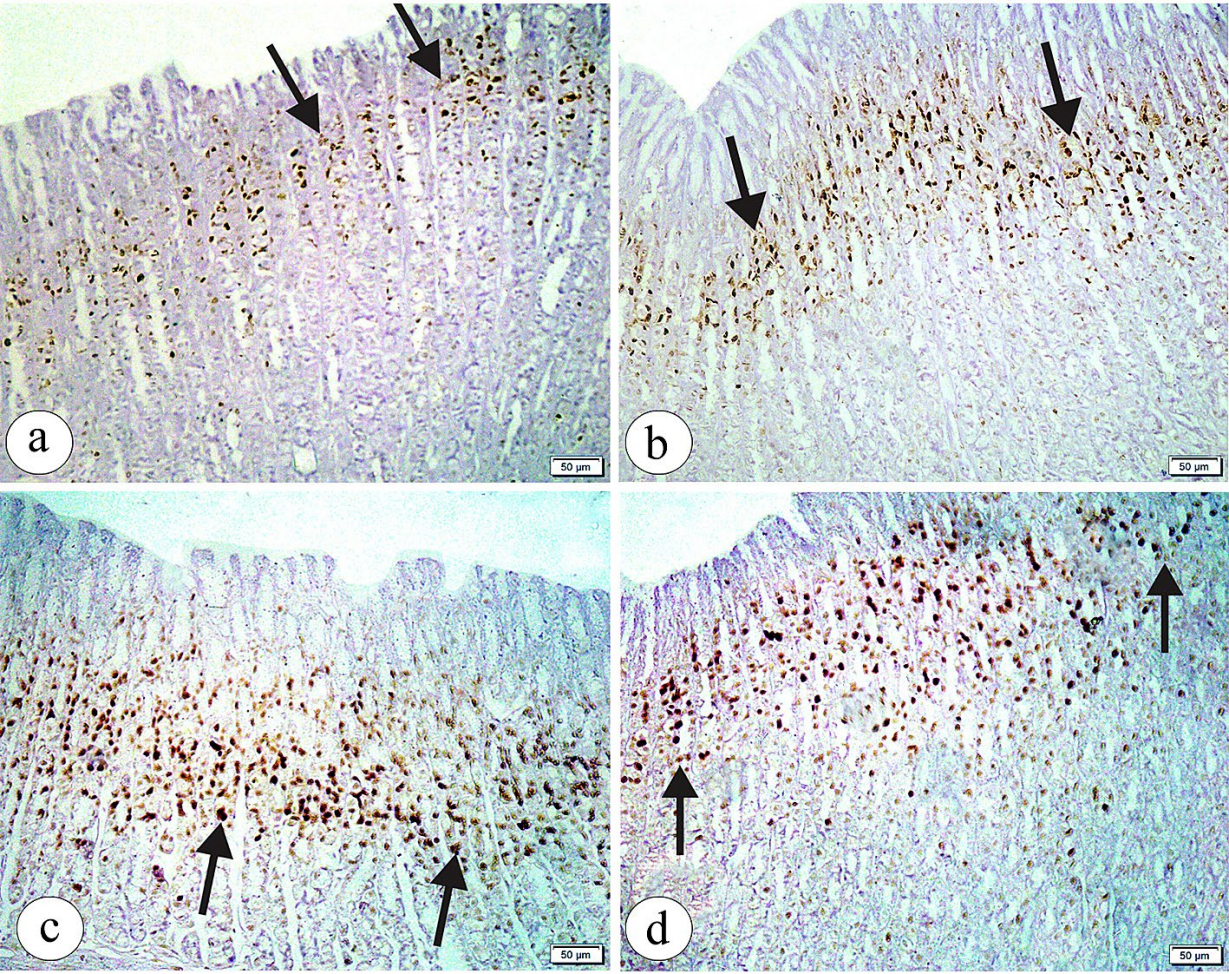
The photomicrographs of the sections in the glandular portion of the stomach in (**a**) the group Ӏ showing a positive PCNA immunoexpression (arrow) in the gastric cells’ nuclei of the upper gastric glands. (**b**) the group ӀӀ showing a positive PCNA immunoexpression(arrow) more or less than the group Ӏ. (**c**) the group ӀӀӀ showing an apparently increased positive PCNA immunoexpression (arrow)in the gastric cells’ nuclei extending into the basal part of the gastric glands as compared to the group Ӏ (**d**) the group ӀV showing an apparent decrease in the positive PCNA immunoexpression (arrow) in the gastric cells’ nuclei as compared to the group ӀӀӀ. (PCNA immunohistochemical staining × 200, Scale bar = 50 µm).

A positive PCNA immunoexpression in the gastric cell nuclei of the upper gastric gland areas in both groups Ӏ and ӀӀ was noticed (Fig. 5a, b respectively). Otherwise, group ӀӀӀ showed an apparently increased positive PCNA immunoexpression in the gastric cell nuclei extending into the basal part of the gastric glands as compared to group Ӏ (Fig. 5c). An obvious decrease in the positive PCNA immunoexpression in group ӀV was observed as compared to group ӀӀӀ (Fig. 5d). Quantitative analysis displayed that group ӀӀӀ showed a significant increase in the area percent of PCNA immunoreaction and non-significant increase in the PCNA optical density as compared to control group. However, group ӀV showed a significant decrease in the area percent of PCNA immunoreaction and non-significant decrease in the PCNA optical density as compared to group ӀӀӀ (as showed in Table 2).

**Figure 6:**
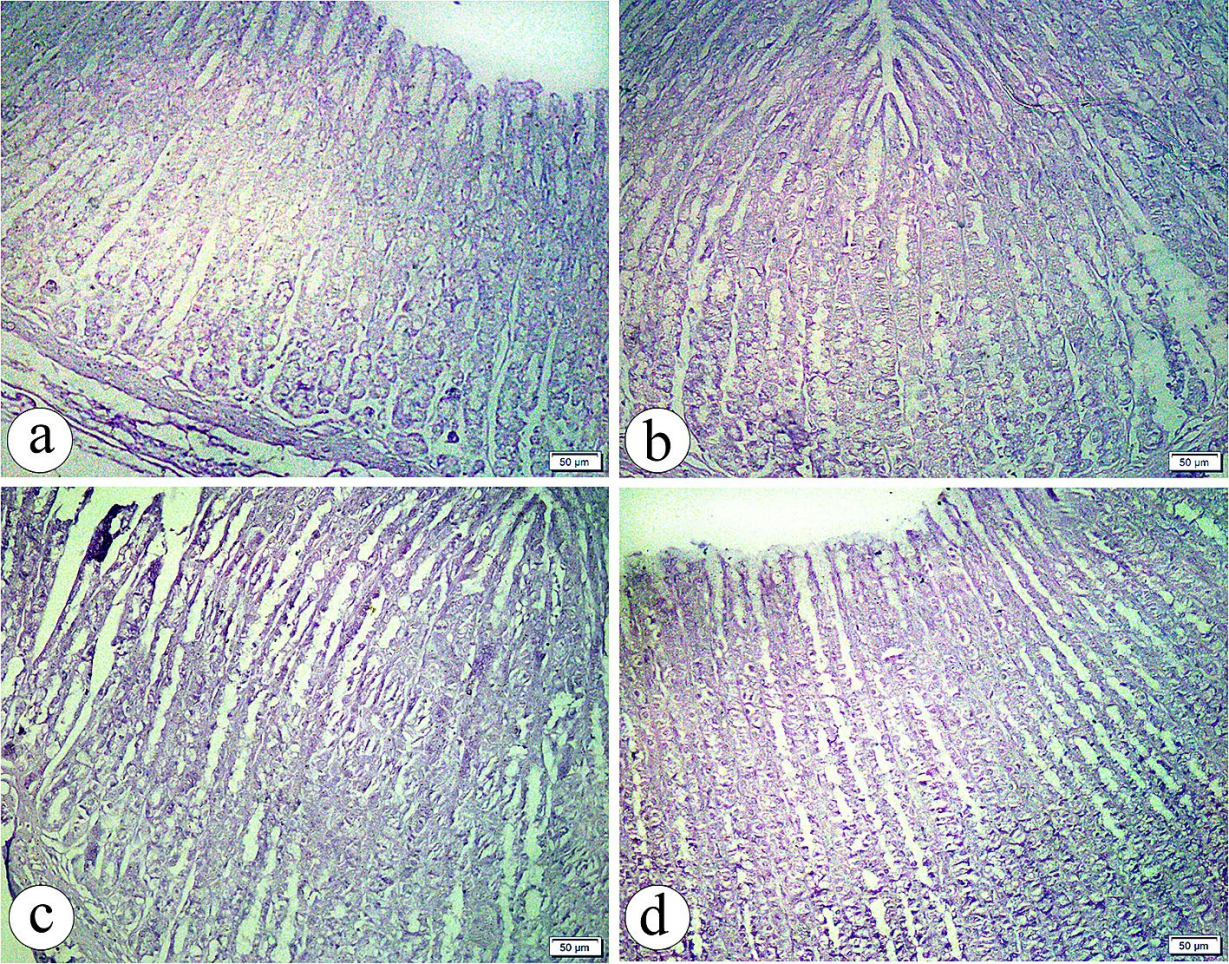
The photomicrographs of the sections in the glandular portion of the stomach in (**a, b, c, d**) showing a negative EGFR immunoexpression in all studied groups; Ӏ, ӀӀ, ӀӀӀ, ӀV, receptively. (EGFR immunohistochemical staining × 200, Scale bar = 50 µm).

Interestingly, a negative EGFR immunoexpression appeared in all studied groups Ӏ, ӀӀ, ӀӀӀ and ӀV (Fig. 6a, b, c, d receptively).

### Electron microscopic study

**Figure 7:**
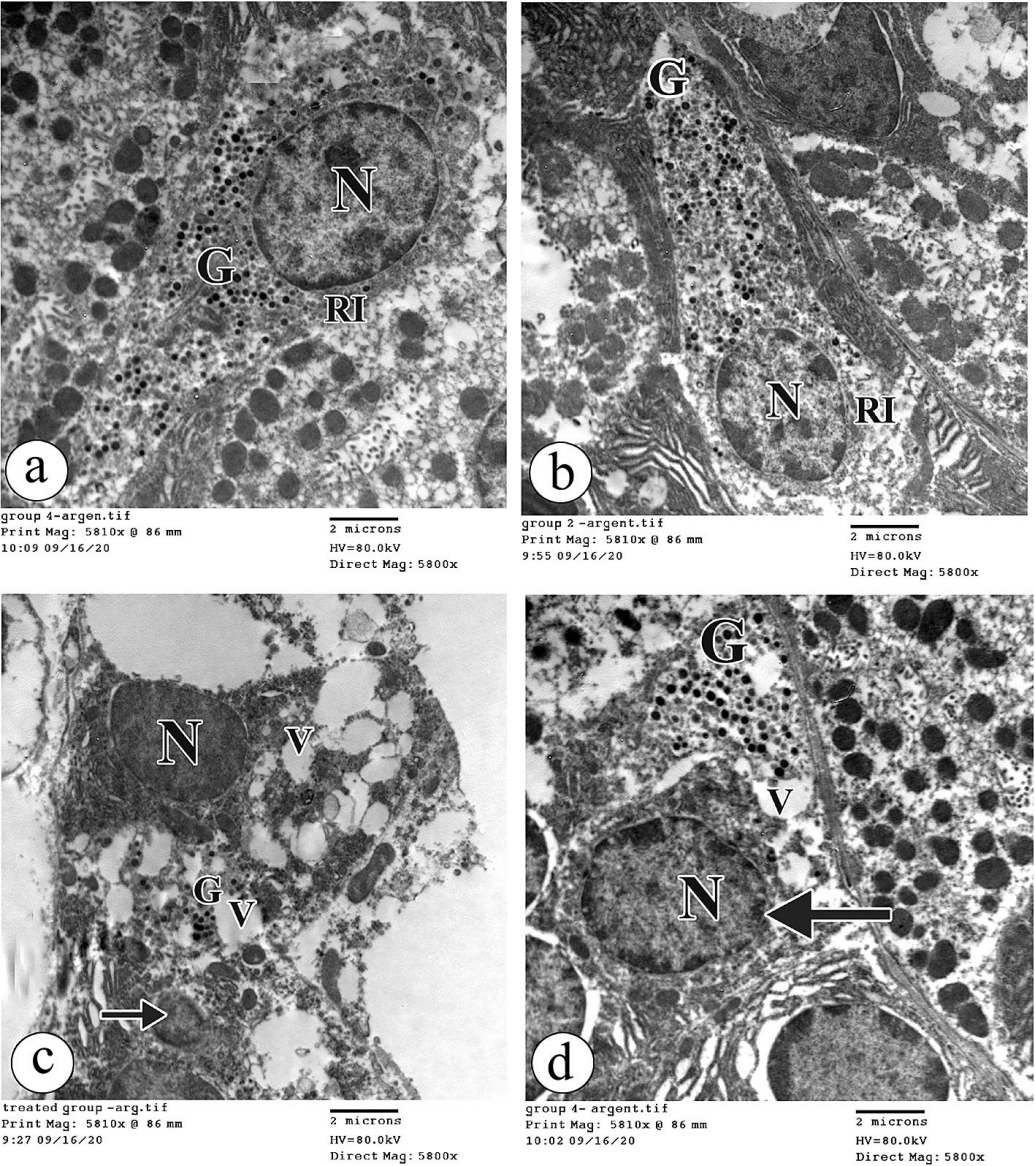
The electron photomicrographs of the enteroendocrine cell in (**a**) the group Ӏ showing an euchromatic nucleus (N) with many tiny electron-dense granules (G) and abundant ribosomes (RI). (**b**) the group ӀӀ showing a similar ultrastructural morphology to that in the group Ӏ. (**c**) the group ӀӀӀ showing an electron dense nucleus (N) and many vacuolations (V). Few tiny electron-dense granules (G) and a loss of cell organelles are noticed. A pyknotic shrunken nucleus (arrow) is seen. (**d**) the group ӀV showing an euchromatic nucleus (N) with a nuclear membrane indentation (arrow). The cytoplasm shows many tiny electron-dense granules (G) and vacuolations (V). (TEM × 5800, Scale bar = 2 µm).

Ultra-structurally, the enteroendocrine cells in both groups Ӏ and ӀӀ had euchromatic nuclei and their cytoplasm contained many tiny electron-dense granules and abundant ribosomes (Fig. 7a, b respectively). On the other hand, in group ӀӀӀ the enteroendocrine cells had some electron dense nuclei and other nuclei appeared pyknotic and shrunken. The cytoplasm had many vacuolations and few tiny electron-dense granules. A marked loss of the cell organelles was noticed (Fig. 7c). Interestingly, the enteroendocrine cells in group ӀV demonstrated euchromatic nuclei with nuclear membrane indentations. The cytoplasm had many tiny electron-dense granules and vacuolations (Fig. 7d).

**Figure 8:**
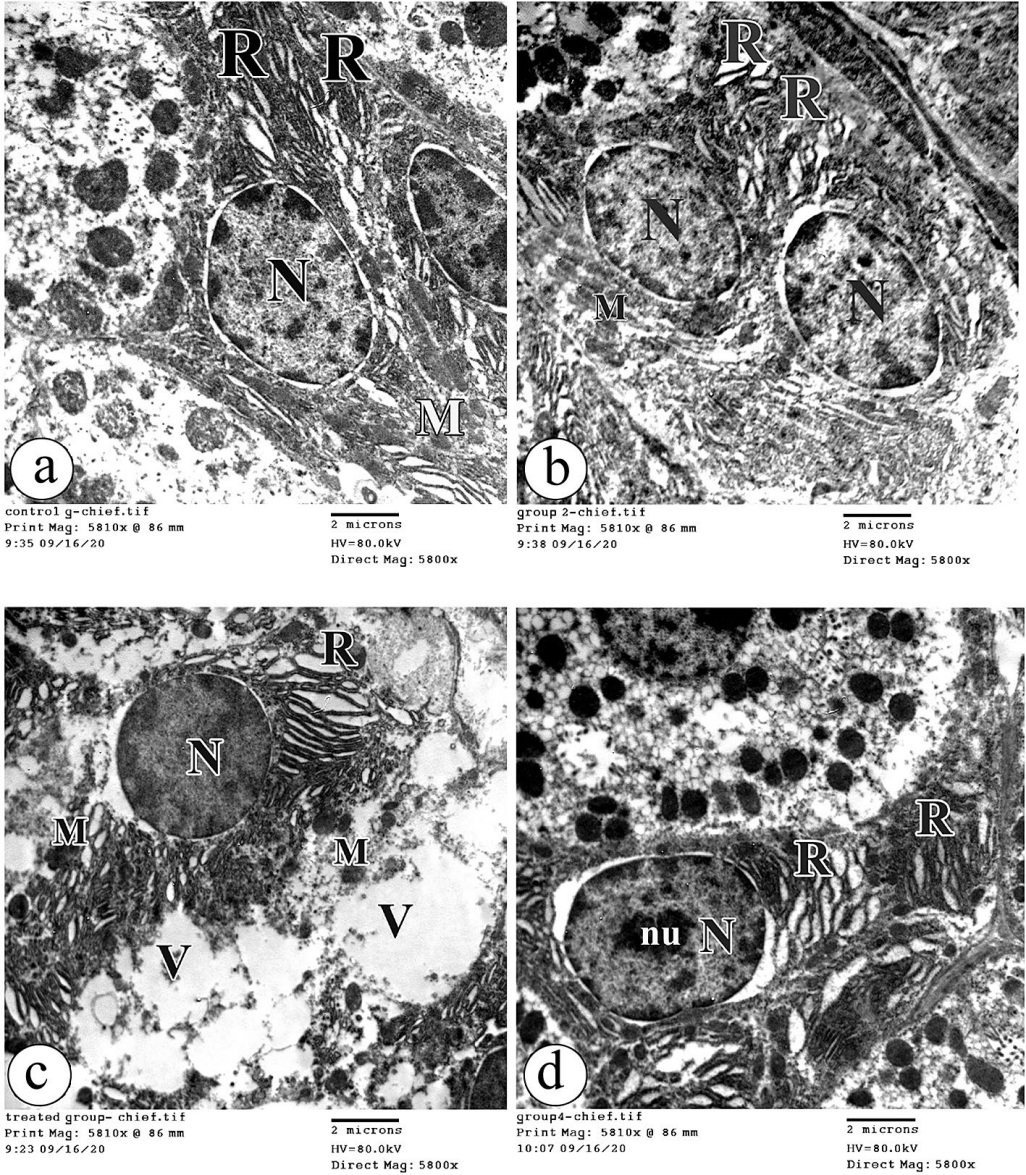
The electron photomicrographs of the Chief cell in (**a**) the group Ӏ showing an euchromatic nucleus (N), mitochondria (M) and an abundant packed rough endoplasmic reticulum (R). (**b**) the group ӀӀ showing an ultrastructural morphology as in the group Ӏ. (**c**) the group ӀӀӀ showing an electron dense nucleus (N), electron dense mitochondria (M) and many vacuolations (V). Dilatation and elongation of the rough endoplasmic reticulum (R) are observed. (**d**) the group ӀV showing an electron dense nucleus (N) with nucleolus(nu) and a dilated rough endoplasmic reticulum (R). (TEM × 5800, Scale bar = 2 µm).

Regarding Chief cells in both groups Ӏ and ӀӀ, they had euchromatic nuclei. The cytoplasm contained mitochondria and abundant packed rough endoplasmic reticula (Fig. 8a, b respectively). With BPA treatment in group ӀӀӀ, Chief cells contained electron dense nuclei, electron dense mitochondria and many vacuolations. Moreover, dilatation and elongation of the rough endoplasmic reticulum were observed (Fig. 8c). With the concomitant BPA + Curcumin administration in group ӀV, Chief cells appeared with electron dense nuclei and nucleoli. Dilated rough endoplasmic reticula were noticed (Fig. 8d).

**Figure 9:**
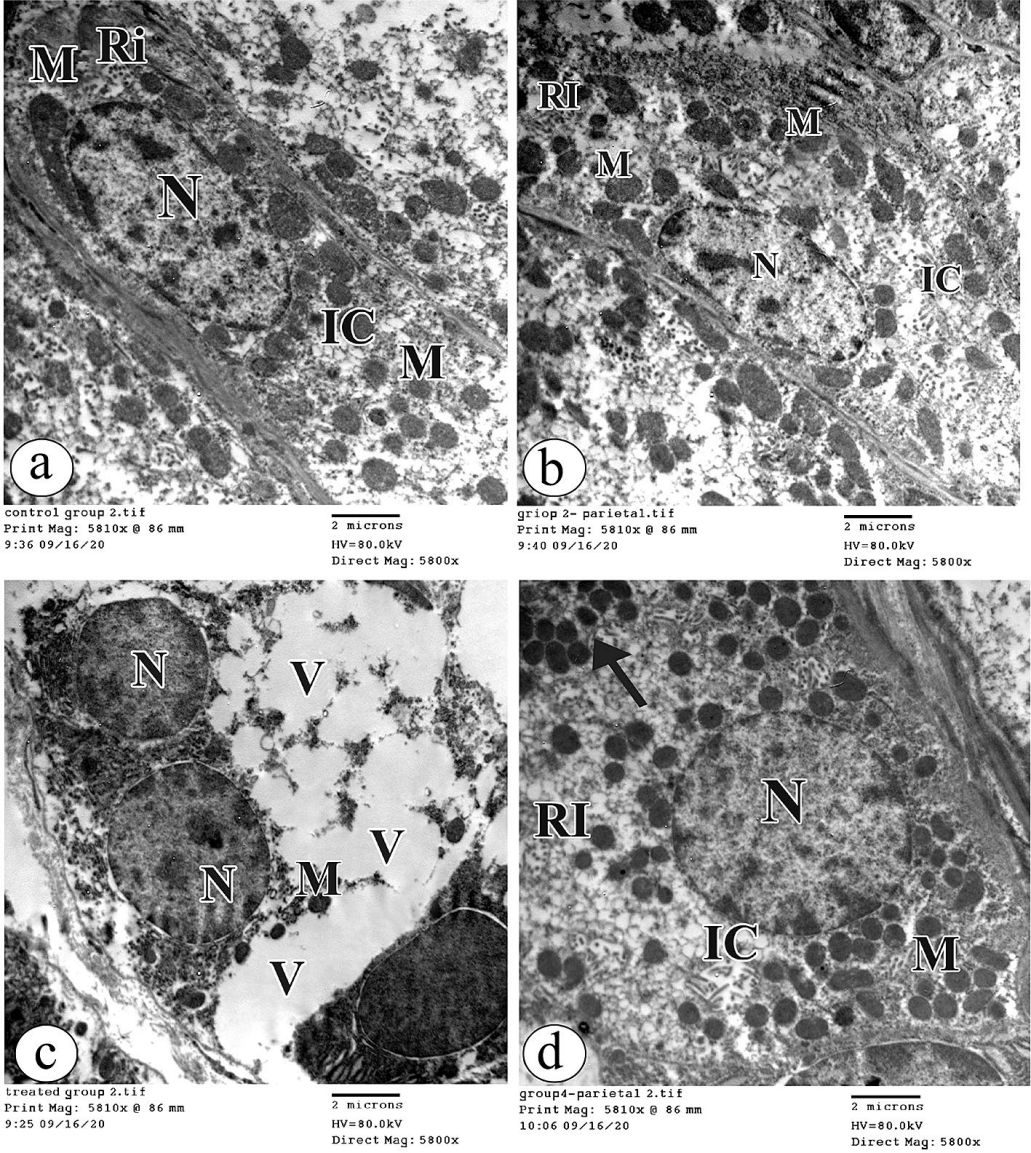
The electron photomicrographs of the parietal cell in (**a**) the group Ӏ showing an oval euchromatic nucleus (N). The cytoplasm contains abundant mitochondria (M), intracellular canaliculi (IC) and free ribosomes (RI). (**b**) the group ӀӀ showing an ultrastructural morphology as in the group Ӏ. (**c**) the group ӀӀӀ showing a marked loss of cell organelles with a vacuolated cytoplasm (V). Few electron dense mitochondria (M) and another destructed one (arrow) are noticed. The euchromatic nucleus (N) is seen. (**d**) the group ӀV showing an oval euchromatic nucleus (N). The cytoplasm contains the intracellular canaliculi (IC) and free ribosomes (RI). Some mitochondria (M) appear normal; however, others are electron dense (arrow). (TEM × 5800, Scale bar = 2 µm).

The parietal cells in both groups Ӏ and ӀӀ had oval euchromatic nuclei. The cytoplasm contained abundant mitochondria, intracellular canaliculi, and free ribosomes (Fig. 9a, b respectively). On the contrary, the parietal cells in group ӀӀӀ showed a marked loss of the cell organelles with a vacuolated cytoplasm. Few electron dense mitochondria and other destructed were detected. Also, the euchromatic nuclei were observed (Fig. 9c). Impressively, the parietal cells in group ӀV had oval euchromatic nuclei. The cytoplasm contained the intracellular canaliculi and free ribosomes. Some mitochondria appeared normal; however, others were electron dense (Fig. 9d).

## Discussion

BPA is widely used as a major component of manufacturing plastics, raising many considerations about its health impact on humans. BPA’s size and structure are like estradiol and act on estrogen receptors, disturbing the endocrine function. Therefore, many studies have been conducted on low-dose BPA exposure, showing the toxic effects of BPA on the neurological, cardiac, and renal tissues as well as the male and female reproductive systems^27–30^.

After ingesting products packed in plastic containers, BPA may be absorbed in the gastrointestinal tract, conjugated by glucuronic acid in the intestine and liver, then excreted as BPA glucuronide in urine within 24 h^31^. Moreover, BPA was found in the stomach of rats after an IV injection of 0.73 mg/kg dose. In this study, the chosen BPA dose was based on the not observable adverse effect levels recorded in the rat and mouse; 50 mg/kg/d^32^. The standard daily tolerable BPA dose for humans is 50 μg/kg/day^30^. Nevertheless, the ‘safe’ dose of BPA remains debatable^33^.

Curcumin exhibits numerous protective impacts and an increase in mucosal defensive mechanisms on the gastrointestinal tract, such as inhibiting intestinal spasm and ulcer formation caused by alcohol, stress, and drugs^14,34^. Also, curcumin plays a key role in patients with precancerous lesion improvement and inhibits carcinogenesis in intestinal metaplasia, glandular stomach precancerous lesion, cervical intraepithelial neoplasm, oral leukoplakia, and bladder cancer^35,36^. Furthermore, curcumin has been used in malignant, degenerative neurologic diseases, such as Alzheimer's disease and Parkinson’s disease, cardiovascular diseases, and inflammatory disease therapy^15,37^. Curcumin has a rapid metabolism and fast elimination, and the stomach is its most likely absorption site. This is because curcumin’s serum concentration becomes high 3 h post-feeding^38^.

Curcumin is considered a ‘novel drug’ with great potential and is marketed as a supplement in numerous forms, such as tablets, ointments, energy drinks, and cosmetics^39^.

Gastric cancer is the third leading cause of death among cancers and the most frequent malignancies recorded globally. Ingesting food containing carcinogens is a risk factor for precancerous lesion development^40^. However, limited data have been published on the effects of BPA on the gastrointestinal tract. Until now, this study is the first to consider the impact of BPA on the structure of the glandular portion of the stomach in adult albino rats. Also, we looked at the effects of BPA on the structure of the stomach and the possible role of curcumin. Our study is mainly descriptive and limited to morphological and histological data and further biochemical and genetics studies are needed.

Furthermore, light microscopy of the gastric sections showed that BPA administration destroyed gastric glands. Parietal cells had dense nuclei and cytoplasm, and chief cells revealed cytoplasmic vacuolation. Furthermore, dilated congested blood vessels and submucosal edema were observed. A previous study showed that edema with BPA administration might be related to histamine release following gastric damage that led to vasodilatation and a rise in capillary permeability and interstitial fluid^41^.

The dilated congested blood vessels observed in this research agree with previous studies, that detected BPA-induced severe vascular congestion in the heart^7^ and lung^42^.

Surprisingly, with curcumin treatment, a partial restoration of the normal histological structure of the glandular portion of the stomach was noticed. Similarly, a study by^43^ showed that curcumin induced a protective impact on the stomach morphology in rats due to the absolute ethanol degenerative effect and improved the congestion of the gastric blood vessels.

In the negative and positive control groups, PAS-stained sections demonstrated a normal, strong positive reaction in the lining epithelium and neck cells of the glandular portion of the stomach. Interestingly, a decrease in PAS-positive reactivity and depletion in mucin was observed in the BPA-administered group. The findings of this study agree with those of^30^, showing a weak PAS-positive reaction in the prostate of rats treated with BPA. Since the mucus protects the gastric mucosa from oxidative damage attributable to its gel criteria**,** a decrease in the gastric mucus increases the possibility of gastric mucosal damage^44^. In contrast, in this study with curcumin co-administration, PAS-stained sections showed restoration of the positive PAS reaction**.** These findings indicated that curcumin had a protective effect on the gastric mucous membrane by increasing gastric wall mucus production. This suggestion agrees with the conclusion of ^14^ that curcumin prevents ulcer formation by significantly increasing the gastric mucus in rats exposed to gastric insults.

Also, in this study, gastric sections showed a normal distribution of collagen fibers among the bases of the gastric glands and in the lamina propria in both control groups. However, BPA elicited increased collagen fiber deposition. Surprisingly, minimal interstitial collagen fiber deposition was noticed with curcumin treatment. These findings agree with the preceding studies that found BPA-induced liver^45^, myocardial^1^, prostatic^30^, and lung fibrosis^42^.

The increase in fibroblast activity might cause gastric fibrosis, and different cell types stimulated by epithelial cells and basement membrane injury, leading to collagen production in response to BPA^42^.

Furthermore, curcumin administration in this study appeared to reduce collagen deposition related to its anti-inflammatory potency. Oral curcumin is helpful in chronic inflammation like steroidal drugs by inhibiting the release of pro-inflammatory cytokines, such as tumor necrosis factor-α, leptin, interferon-γ, and interleukin-1, and reducing cellular infiltration^14^. Additionally, the mechanism of action of curcumin has multiple cellular targets. Curcumin can regulate the expression of pro-inflammatory mediator and antioxidant genes, deactivating the transcription factor nuclear factor-κB (NF-κB) by blocking I-κB phosphorylation and I-κB kinase complex inactivation^46^. Also, curcumin can stop the activator protein-1AP-1 because it has a direct connection to AP-1 DNA binding and also stops c-Jun and c-fos from binding to it^17^.

The degenerative findings of BPA observed in this study agree with a previous study on renal tubular lining cells^6^ and hepatocytes^3^. In addition, the antifibrotic mechanism of curcumin might also be related to its capability to decrease the expression of TGF-β and increase the expression of α-smooth muscle actin (α-SMA)^47^.

In this study, signs of apoptosis were detected in most parietal, chief, and enteroendocrine cells of the gastric glands after BPA administration. In addition, the nuclei looked pyknotic and shrunk with heterochromatin condensation, which means that DNA fragmentation happened after BPA was used.

The degenerative changes in the mitochondria in parietal, chief, and enteroendocrine cells observed in this study suggest that the mitochondria are target cell organelles of BPA have an impact on the gastric structure. Furthermore, this hypothesis is supported by a previous study^45^, which reported BPA-induced hepatic mitochondrial dysfunction. The cause of mitochondrial damage might be oxidative stress and accumulated ROS in the mitochondria. In turn, damaged mitochondria led to the manufacturing of more ROS, entering the vicious circle of cell damage. This suggestion is supported by the study by^48^.

However, curcumin treatment in this study significantly improved cell organelles’ morphology, and curcumin is a potent ROS scavenger, which can prevent mitochondrial dysfunction. These suggestions are supported by the study by^49^, who concluded that curcumin co-administration in the animal model of non-steroidal anti-inflammatory drug (NSAID)-induced gastroenteropathy significantly decreased GIT MDA levels and attenuated oxidative stress, preventing mitochondrial dysfunction. Also,^50^ reported that curcumin possessed good ROS scavenging abilities in the stomach of rats and interacted with the DNA of the gastric mucosa. ^51,52^ showed that phenolic, β-diketone, and methoxy groups in the curcumin structure liberate electrons to neutralize the free radicals and are attributable to the ROS scavenging ability of curcumin. In contrast^53^, concluded that curcumin activated the alteration of the membrane potential of mitochondria and discharged cytochrome C, initiating caspase-3 activation.

On ultrastructural evaluation, BPA induced severe damage to the rough endoplasmic retinaculum, which plays a vital role in the protein pathway. These observations suggest that BPA can induce rough endoplasmic reticulum stress-related apoptosis. This hypothesis is supported by the previous work of^54^, which showed that BPA caused elongation and stress-inducing cellular death.

Furthermore, an immunohistochemical study was conducted to elucidate the mechanisms underlying the degenerative effects of BPA on the histological structure of the glandular portion of the stomach. To investigate the involvement of BPA in antiapoptotic Bcl-2 induced apoptosis, BPA treatment reduced Bcl-2 expression compared with the negative and positive control groups. These results support the previous study of^55^, who suggested that the lowered level of the antiapoptotic protein Bcl-2 in germ and Sertoli cells following BPA exposure was considered evidence of germ cell apoptosis through the medium of the mitochondrial and Fas/FasL signalling pathway. Also, these study’s results agree with those of BPA effects in the liver of male rats by^56^. Therefore, the possible mechanism of BPA-induced apoptosis may be attributed to its role in elevating intracellular calcium levels, leading to induced ROS generation, MAPK activation, and a pro-apoptotic cascade, thereby inhibiting cell survival. In addition, BPA induces ROS production and significantly compromises mitochondrial function^57^.

Interestingly, BPA + curcumin administration increased Bcl-2 expression. These results agree with those of a previous work by^25^, who showed that curcumin had a potent antiapoptotic effect by reducing Bax and caspase-3 gene expression and boosting Bcl-XL gene expression. Also, these findings were observed in various organs, such as the testis, lung, and kidney^58–60^.

To assess the possible BPA role in inducing gastric epithelial cell differentiation and proliferation, this study showed a negative EGFR immunoexpression in all study groups proposing no BPA role in stimulating epithelial cell proliferation. EGFR signaling in gastric cells plays a role in gastric injury repair following acute insult and ulcer healing due to the release of variable stimuli, such as TNF-α^23,61^. In contrast, the proliferation of breast cancer cells was detected following BPA exposure, which may be due to an upregulation of cell cycle genes and a downregulation of antiproliferative genes^62^.

Regarding the effect of low-dose BPA on PCNA immunoexpression, this study revealed an increased PCNA immunoreaction in BPA-treated rats. This represents strong evidence that low-dose BPA exposure is a potential risk of neoplastic alteration. Also, this might be related to BPA endocrine disruptor power distorting biological functions and organ structures. These findings agree with the former study^32^, which mentioned that BPA stimulated a significant rise in PCNA expression in the liver, mammary gland, and ovary and could induce hepatic, prostatic, and testicular cancers.

However, a reduced PCNA immunoreaction in BPA + curcumin-treated rats was evident. This agrees with another study^63^, which mentioned that curcumin reduced PCNA immunoreactivity in the uterus and ovary of arsenicated rats. In contrast, curcumin treatment in the study by^64^ significantly raised the number of PCNA-positive germinal cells in the testes of diabetic rats.

Furthermore, BPA administration reduced antiapoptotic protein (BCL2) immunoexpression in the rats’ stomachs. Therefore, it is proposed that mitochondria are target cell organelles of BPA effects. This suggestion agrees with the study by^65^ that showed that mitochondria play an important role in apoptosis by liberating intermembrane space proteins, such as cytochrome C, which activates caspases in the cytosol and mediates apoptosis. Additionally, the mechanism of BPA-induced apoptosis might be due to a disproportion in the expression of proapoptotic to antiapoptotic proteins of the BCL2 family and BCL-2-associated X (BAX) on the outer mitochondrial membrane, releasing apoptogenic factors^45^.

The BPA group showed reductions in body and stomach weights, that were statistically significant. These findings agree with the results of previous studies ^3,5,66,67^, which found that BWs of BPA-treated groups were significantly lower than those of control groups. Also, this reduction might be attributed to the endocrine disruptor BPA action, which created hormone excretion imbalance and metabolic disorders in the form of alterations in serum lipid concentrations and estradiol and steroid production^68^. Moreover^32^, mentioned that the reduction in weight gain was associated with a reduction in food consumption in BPA rats compared with control rats.

In contrast, previous studies by^26^ and^6^ demonstrated no change in the prostate, seminal vesicle, and epididymis, body, relative kidney, liver, and spleen weights in BPA-treated rats compared with control rats. Furthermore^69^, concluded that BPA increased body weight as it disrupted glucose homeostasis, increased glucose transporter 4 proteins in the adipocyte tissue, basal and insulin-stimulated glucose uptake, and decreased adiponectin levels. Moreover, they suggested that BPA contributed to the obesity epidemic, as early BPA exposure could modify developmental programming. Additionally, BPA possessed estrogenic action and stimulated the growth hormone release^70^.

Many mechanisms might explain the degenerative BPA impact on the stomach. First, BPA induces oxidative stress-reducing glutathione-S-transferase (GST), GPx activity, and a rise in MDA levels, interfering with the detoxification of lipid peroxides and downregulation of the gene expression of antioxidant enzymes in different organs. Also, BPA increases NO production, augmenting oxidative stress. Also, oxidative stress triggers the oxidation of cellular molecules (proteins, DNA, and polyunsaturated fatty acids (PUFAs)), inducing marked cellular destruction, cytolysis, and chromosomal degradation.

Secondly, the estrogenic activity of BPA is mediated by endocrine signalling pathways, resulting in large changes in cell functions. Additionally, BPA induces mitochondrial-mediated apoptosis by caspase-3 activation, which is a keystone in inflammation, fibrosis, inflammatory cytokine dysregulation in the form of increasing pro-inflammatory cytokines (IL1b) and decreasing anti-inflammatory/antifibrotic cytokines (IL-10)^3,4,29,45,66,71,72^.

The observed improvement of the gastric histological structure with curcumin concomitant administration may be attributed to its high anti-inflammatory, immunomodulatory, antioxidant, and anticancer activities. Also, it indirectly increases glutathione, glutathione reductase, GST, vitamin C levels, superoxide dismutase (SOD), and catalase (CAT) activities, inducing antioxidant status and preventing lipid peroxidation and DNA damage. Other curcumin targets include EGFR, which suppresses prostaglandin synthesis through its effect on cyclooxygenase (COX), a key enzyme responsible for converting arachidonic acid to prostaglandins. Additionally, curcumin stimulates cytoprotective enzymes, such as heme oxygenase, glutamate-cysteine ligase, and quinone oxidoreductase 1. Moreover, curcumin promotes cell signalling and possesses significant chemopreventive activity^14,39,43,51,73^.

## Conclusion and recommendations

Conclusively, BPA exposure has toxic effects on the glandular portion of rats’ stomachs. BPA exposure destroyed gastric glands; parietal cells had dense nuclei and chief cells, revealed vacuolated cytoplasm. Also, dilated congested blood vessels, submucosal edema, and collagen deposition were observed. BPA administration induced a decreased PAS-positive reactivity and BCL2 immunohistochemical expression. Furthermore, BPA-induced DNA damage is demonstrated by an increase in PCNA immunoexpression. Moreover, the BPA group showed a significant reduction in the final body and stomach weights and an insignificant reduction in the gastric mucosal heights.

Curcumin-treated rats displayed a noticeable improvement in the structure of the glandular portion of the stomach associated with an increase in the PAS reaction and BCL2 immunohistochemical expression and a decrease in PCNA immunohistochemical expression and collagen deposition. Moreover, a significant increase in the final body and stomach weights and an insignificant rise in the gastric mucosal heights were found.

Thus, based on accumulating evidence, BPA exposure is harmful to the glandular portion of the stomach due to its fibrotic and apoptotic properties. However, an obvious protective role of curcumin due to its antifibrotic and antiapoptotic properties is established, delivering a promising natural protective effect against environmental and occupational toxins. Therefore, it is recommended that the extensive use of BPA-containing plastic products should be avoided, and other safe alternative products should be used. Furthermore, workers in plastic factories should be protected and undergo a periodic follow-up. Additionally, further studies should be performed to determine the molecular and genetic effects of BPA.

## Data Availability

Available from corresponding author upon reasonable request.
